# To test or not to test: A cross-sectional survey of the psychosocial determinants of self-testing for cholesterol, glucose, and HIV

**DOI:** 10.1186/1471-2458-11-112

**Published:** 2011-02-17

**Authors:** Janaica EJ Grispen, Gaby Ronda, Geert-Jan Dinant, Nanne K de Vries, Trudy van der Weijden

**Affiliations:** 1 Department of General Practice, Faculty of Health, Medicine, and Life Sciences, Maastricht University, Maastricht, The Netherlands; 2 Department of Health Promotion, Faculty of Health, Medicine, and Life Sciences, Maastricht University, Maastricht, The Netherlands; 3 CAPHRI School for Public Health and Primary Care, Maastricht University, Maastricht, The Netherlands

## Abstract

**Background:**

Although self-tests are increasingly available and widely used, it is not clear whether their use is beneficial to the users, and little is known concerning the determinants of self-test use. The aim of this study was to identify the determinants of self-test use for cholesterol, glucose, and HIV, and to examine whether these are similar across these tests. Self-testing was defined as using in-vitro tests on body materials, initiated by consumers with the aim of diagnosing a particular disorder, condition, or risk factor for disease.

**Methods:**

A cross-sectional Internet survey was conducted among 513 self-testers and 600 non-testers, assessing possible determinants of self-test use. The structured questionnaire was based on the Health Belief Model, Theory of Planned Behavior, and Protection Motivation Theory. Data were analyzed by means of logistic regression.

**Results:**

The results revealed that perceived benefits and self-efficacy were significantly associated with self-testing for all three conditions. Other psychosocial determinants, e.g. gender, cues to action, perceived barriers, subjective norm, and moral obligation, seemed to be more test-specific.

**Conclusions:**

Psychosocial determinants of self-testing are not identical for all tests and therefore information about self-testing needs to be tailored to a specific test. The general public should not only be informed about advantages of self-test use but also about the disadvantages. Designers of information about self-testing should address all aspects related to self-testing to stimulate informed decision making which, in turn, will result in more effective self-test use.

## Background

A range of self-tests for more than 25 conditions, ranging from infectious diseases to cardiovascular diseases, has become available to consumers in the Netherlands and elsewhere [1]. We defined self-tests as in-vitro tests on body materials such as blood, urine, faeces, or saliva that are initiated by consumers to diagnose a particular disorder or risk factor. Four types of self-tests can be distinguished that are directly available to consumers without the need to consult a physician first [1]. The first type comprises the true self-tests, the over-the-counter tests, in which the consumer is responsible for execution, interpretation, and follow-up behaviour. The second type, the so-called street-corner tests, are in most cases offered by an organization which conducts the test in local supermarkets, and the results are immediately available. The third and fourth self-test categories include direct-access laboratory tests and home-collect or direct-to-consumer tests, in which a consumer attends a laboratory facility to have body material taken or sends body material to a laboratory where the test is done, after which the result is sent to them by mail or via the Internet.

A 2006 Internet survey of the prevalence of self-testing showed that 16% of a sample of Dutch Internet users had ever used a self-test, whereas 17% of non-testers indicated they intended to use a self-test in the future [1]. Self-testing seems ideal as it fits in with the right to self-determination. Individuals take responsibility for their own health by means of self-diagnosis, which is in line with current views on patient autonomy and self-management [2,3]. However, the value of self-tests has been heavily debated in the scientific literature. Proponents argue that self-testing increases testing rates, resulting in more timely diagnosis and treatment, self-testing is convenient and provides anonymity, and it promotes patient empowerment [4-7], while opponents hold that at-risk populations do not use self-tests, self-tests entail relatively high costs, self-tests can be used without assessing the whole clinical picture, and testing without counselling may result in adverse psychological outcomes [4-6,8]. Additionally, self-testing is one of many possible tools to diagnose a certain disease or risk factor and if used outside at-risk populations the possibility of false-positive and false-negative results increases [2,8]. In other words, it is unclear whether self-tests are used in a way that is advantageous to the users [5]. Therefore, effective consumer education seems essential in this new area, as self-tests are likely to become even more easily available and more widely used [2]. However, before we can consider educating consumers about self-testing, it is important to know which factors contribute to the use of self-tests, in order to tailor education to these factors. This should result in information and education stimulating informed decision making and consequently in more effective use of self-tests [9]. To the best of our knowledge, however, no research to date has examined the psychosocial determinants of self-test use. In this study, we made a first attempt to investigate the psychosocial determinants of self-test use, specifically for cholesterol, glucose, and HIV tests, among a sample of Dutch Internet users, and to examine whether these determinants are similar across the different tests considered.

This paper focuses on self-tests for cholesterol, glucose and HIV for the following reasons. All three tests are frequently used in the Netherlands, 38.6%, 34.1% and 11.1% of all self-testers who indicated having ever used a self-test performed a cholesterol, glucose, or HIV self-test, respectively [1]. On the other hand, they differ in terms of the seriousness of the disorder, varying from a risk factor to a sexually transmitted (chronic) infection with serious consequences for the patient's medical prognosis and social life. Furthermore, cholesterol and glucose self-tests are relevant true home self-tests, while the results of HIV self-tests are usually analysed in a laboratory [1]. We chose to examine whether the psychosocial determinants of self-testing differ between those three frequently used, but in several aspects different self-tests, because effective consumer information needs to be tailored to the determinants that are related to the specific self-test and to the self-test type.

Since little is known about the psychosocial determinants of self-test use a broad spectrum of possible psychosocial determinants was assessed. A questionnaire was developed, based on the Health Belief Model (HBM) [10] as well as several concepts of the Theory of Planned Behaviour (TPB) [11] and the Protection Motivation Theory (PMT) [12]. The HBM was originally designed to explain relatively simple health behaviours, such as screening, which may be considered similar to self-testing [10,13,14]. The HBM states that health-related behaviour is based on an individual's perception of the susceptibility to and the severity of a particular condition or illness and the individual's belief that a particular action would reduce their susceptibility to or the severity of this condition. However, action will only be taken if the perceived barriers to the behaviour are outweighed by the perceived benefits and if there are cues (e.g. bodily or environmental events) that trigger action. In 1988, the self-efficacy concept was added to the HBM [10,13,15].

Several additional concepts of the TPB and the PMT were used in this study, such as subjective norm, anticipated regret, moral obligation, and response efficacy. These factors have been shown to contribute to the explanation of health-related behaviour [16-21]. Table 1 provides an overview of constructs, conceptual definitions, and the items that measured the constructs. The aim of this study was to identify the determinants of self-test use and to determine whether these are similar across different tests.

**Table 1:** Overview of constructs, conceptual definitions, items, and answering options

Constructs and definition
Items/answering options^a/b^
**Perceived susceptibility**: the individual's belief of the chance of contracting a certain disease/condition
1. According to you, what are the chances that you will develop elevated cholesterol levels? Answering options: 1 = *very low - *5 = *very high*
2. According to you, what are the chances that you will develop elevated cholesterol levels compared to others of your age and gender? Answering options: 1 = *much smaller - *5 = *much larger*
**Perceived severity**: the individual's belief of the seriousness of a certain disease/condition
1. According to you, how severe is an elevated cholesterol level? Answering options: 1 = *not severe at all *- 5 = *very severe*
**Cues to action**: bodily or environmental events that trigger action such as education, symptoms, media
1. Do you or someone in your immediate environment have elevated cholesterol levels? Answering options: 0 = *no*, 1 = *yes*
**Perceived benefits**: the individual's belief that a certain action will effectively reduce the disease threat
1. According to me, performing a self-test is important
2. Self-testing means taking responsibility for your own health
3. Self-testing provides a sense of security about your own health
4. An important advantage of this self-test is a fast result
5. An important advantage of this self-test is privacy
6. An important advantage of this self-test is that it saves time
7. By testing myself, I can reassure myself
8. By testing myself I take care of my own health
9. It feels good to take responsibility for my own health
**Perceived barriers**: the individual's belief about the negative aspects/costs of a specific health action
1. The costs of this self-test are a barrier to me
2. Testing myself would make me too concerned with my health
3. Being (too) much concerned with my health scares me
4. Just thinking about self-testing scares me
5. Just thinking about self-testing makes me insecure
**Self-efficacy**: the individual's confidence in one's capability to successfully perform a certain action (Recoded: 1 = *completely agree - *5 = *completely disagree*)
1. Performing this self-test is difficult
2. When performing this self-test I would miss professional assistance
3. When interpreting the test result I would miss professional assistance
**Subjective norm**: the individual's belief that a certain individual or group support or reject performing that specific action
1. My partner (or others in my immediate environment) expects me to perform this self-test
**Anticipated regret**: the individual's fear of the feeling of regret if a certain action is not performed
1. I would regret it if I didn't perform this self-test and it subsequently appeared that I have an elevated cholesterol level.
**Moral obligation**: the individual's belief of being morally obliged to perform that action
1. I perceive it as a moral obligation to myself to perform this self-test
2. I perceive it as a moral obligation to the people around me to perform this self-test
**Response efficacy**: the individual's belief in the effectiveness of a response to control the risk of a certain disease/condition
1. The result of this self-test is reliable
2. If the test result is normal (nothing's the matter), you can be sure that this result is correct
3. If the test result is abnormal (something's the matter), you can be sure that this result is correct
4. If the test result indicates that something's the matter, I'm able to take the correct subsequent action

^a ^Examples are provided for cholesterol testers. For glucose testers the word 'cholesterol' is replaced by 'glucose'. For HIV testers 'develop elevated cholesterol levels' is replaced by 'become infected with HIV', 'how severe is an elevated cholesterol level' by 'how severe is HIV, and 'have elevated cholesterol levels' by 'have HIV'.

^b ^Unless stated otherwise, items were measured on a 5-point Likert scale ranging from 1 = *completely disagree *to 5 = *completely agree*.

## Methods

### Ethical approval

The Medical Ethical Committee of Maastricht University indicated that no ethical approval was needed for this study.

### Participants and procedure

A cross-sectional survey was conducted by a Dutch ISO-certified institute for online research (ISO 26361 and 20252), named Flycatcher, which was in charge of the recruitment of participants and the distribution of the questionnaire. Their Internet panel consists of Dutch-speaking individuals aged 12 years or older who have an e-mail address. People can apply for the panel via the Flycatcher website (http://www.flycatcher.eu). Various channels are used to recruit new panellists, e.g. mailing lists of third parties (after permission) and word-of-mouth advertising. Panellists received an e-mail containing an invitation to fill out the questionnaire, a link to the questionnaire, and an expiration date. After one week, a reminder was sent to panellists who have not filled out the questionnaire. Based on the length of the questionnaire, panellists received a certain amount of credits for filling out the questionnaire. If a certain amount of credits is earned, panellists can exchange their credits for an actual incentive (e.g. coupons for online shops, books, or theatres).

**Figure 1:**
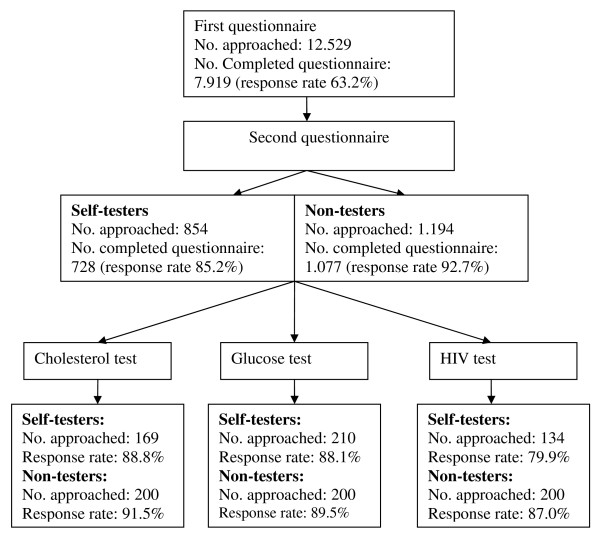
**Flowchart of the questionnaires**. This figure depicts the distribution of the participants divided over the questionnaires regarding the three tests under consideration (cholesterol, glucose, and HIV).

In the current study, two consecutive questionnaires were used. Based on the results of this first questionnaire [1], a questionnaire on the psychosocial determinants of a specific self-test was sent to a selection of self-testers and non-testers. Self-testers received a questionnaire on the test they had performed (e.g. cholesterol test). If multiple self-tests had been performed, a hierarchical selection procedure was applied to determine which test-specific questionnaire was sent, by (1) selecting all respondents who indicated having performed an HIV-test, (2) selecting all respondents who indicated having performed a glucose test and who were not included in (1), and (3) selecting all respondents who indicated having performed a cholesterol test and who were not included in (1) or (2). In addition, a random sample of non-testers was selected based on their level of intention to perform a specific self-test. This resulted in a sample of non-testers equally distributed over the different tests and the various intention categories. Figure 1 displays a schematic overview of the numbers of respondents and non-respondents for each questionnaire.

### Measures

In the current study, two consecutive questionnaires were used. An initial short questionnaire was sent to the full Internet-panel, consisting of 12,529 panelists. This questionnaire was aimed at determining the prevalence of the use of self-tests, types of self-tests, and at providing an overview of demographic characteristics. The results of the first questionnaire are reported elsewhere [1].

After two months, a second questionnaire on the psychosocial determinants of a specific self-test was sent to self-testers and non-testers. Self-testers received a questionnaire on the test they had performed and non-testers received a questionnaire on the test they intended to perform in the future, as indicated in the first questionnaire (e.g. a cholesterol test). The questionnaire assessed the possible determinants of self-test use and was based on the HBM [14], TPB [11], and PMT [12]. The questionnaire measured perceived susceptibility and severity, cues to action, perceived benefits and barriers, self-efficacy, subjective norm, anticipated regret, moral obligation, and response efficacy. All respondents received a comparable questionnaire, tailored to the test performed (e.g. cholesterol testers received a questionnaire about the cholesterol test). *Perceived susceptibility *was measured with two items which were combined to form the susceptibility factor (*M = *2.61, *SD *= .91, with Cronbach's α of. 74, .80, and .64 for cholesterol, glucose, and HIV, respectively). *Perceived severity *was measured with one item (*M = *4.32, *SD *= .69), as was *cues to action*. *Perceived benefits *were measured using nine items which were combined to form the perceived benefits factor (*M = *3.49, *SD *= .52, with Cronbach's α of .77, .79, and .78 for cholesterol, glucose, and HIV, respectively), whereas *perceived barriers *were measured with five items which were combined to form the perceived barriers factor (*M = *2.48, *SD *= .67, with Cronbach's α of .72, .76, and .74 for cholesterol, glucose, and HIV, respectively). *Self-efficacy *was measured with three items which were combined to form the self-efficacy factor (*M = *3.04, *SD *= .84, with Cronbach's α of .63, .76, and .63 for cholesterol, glucose, and HIV, respectively). *Subjective norm *(*M = *2.18, *SD *= .96) and *anticipated regret *(*M = *3.41, *SD *= 1.09) were each assessed with one item. *Moral obligation *was measured using two items which were combined to form the moral obligation factor (*M = *2.84, *SD *= .98, with Cronbach's α of .87, .85, and .90 for cholesterol, glucose, and HIV, respectively). *Response efficacy *was measured with four items which were combined to form the response efficacy factor (*M = *3.70, *SD *= .69, with Cronbach's α of .81, .78, and .82 for cholesterol, glucose, and HIV, respectively). Table 1 provides an overview of constructs, conceptual definitions, items, and answering options.

### Statistical analysis

Analyses were conducted using SPSS 16.0. All analyses described below were conducted separately for cholesterol, glucose, and HIV tests. Unless indicated otherwise, an alpha of .05 was used for statistical significance. First, basic descriptive statistics were used to describe the respondents' socio-demographic characteristics. Multiple logistic regression analyses were then performed, with being a tester or a non-tester as the outcome variable (non-testers = 0; testers = 1), and socio-demographic characteristics (age, gender, and level of education), perceived susceptibility and severity, cues to action, perceived benefits and barriers, self-efficacy, subjective norm, anticipated regret, moral obligation, and response efficacy as potential predictors. Additionally, independent samples T-tests were performed at item level for each construct, with being a tester or a non-tester as the group variable. Because of multiple testing, Bonferroni correction was applied and an alpha of .001 was used for statistical significance.

## Results

### Participant characteristics

The questionnaire was sent to a total of 513 self-testers and 600 non-testers, approximately equally distributed between cholesterol, glucose, and HIV tests, with a response rate ranging from 79.9% to 91.5% (Figure 1). Respondents were between 12 and 94 years of age. Analyses revealed no differences between types of test used and therefore we decided to combine all test-types. The remainder of the results section will only report on the analyses across test-types. Table 2 presents socio-demographic characteristics of the respondents and types of tests performed.

**Table 2:** Socio-demographic characteristics of respondents

Characteristics		Cholesterol		Glucose		HIV	
		Testers	Non-testers	Testers	Non-testers	Testers	Non-testers
**N**		150	183	185	179	107	174
**Age**	*Mean*	37.9	41.3	42.0	29.3 (738.3	29.3 (729.3	29.3 (732.1
	*(SD)*	(14.6)	(13.6)	(13.1)	(13.5)	(7.5)	(12.5)
	*(range)*	(15-82)	(12-94)	(15-79)	(15-81)	(19-61)	(15-70)
**Gender**	*Male*	36.7%	40.4%	27.6%	29.6%	26.2%	37.4%
	*(N)*	(55)	(74)	(51)	(738(53)	(28)	(65)
	*Female*	63.3%	59.6%	72.4%	70.4%	73.8%	62.6%
	*(N)*	(95)	(109)	(134)	(126)	(79)	(109)
**Level of education^a^**	*Low*	13.3%	18.6%	23.2%	17.9%	7.5%	12.6%
	*Intermediate*	42.7%	35.5%	45.4%	33.5%	38.3%	49.4%
	*High*	44.0%	45.9%	31.4%	48.6%	54.2%	37.9%
**Type of self-test used**	*Home test*	35.0%		33%		6.5%	
	*Street-corner test*	51.3%		17.3%		6.5%	
	*Direct-access laboratory test*	9.3%		15.1%		64.0%	
	*Home-collect test*	1.2%		0.5%		1.9%	
	*Different*	3.2%		34.1%		21.1%	

^a ^Low = primary and secondary school, Intermediate = intermediate vocational education, high = higher vocational education and university

### Predictors of self-test use

#### Cholesterol test

Cholesterol testers were more likely to be female than male. Cholesterol testing was also significantly associated with perceived susceptibility, perceived benefits, self-efficacy, and moral obligation. However, cholesterol testers were more likely to perceive fewer barriers to self-testing and to have a lower level of response efficacy than non-testers (Table 3).

**Table 3:** Predictors of self-test use

Variable	Cholesterol test^a^	Glucose test^b^	HIV test ^c^
	*OR*	*OR*	*OR*
	*[95% CI]*	*[95% CI]*	*[95% CI]*
**Demographics**			
Gender	**2.4**	1.2	1.6
	[1.2-4.8]*	[0.6-2.8]	[0.6-4.0]
Age	1.0	1.0	1.0
	[1.0-1.0]	[1.0-1.0]	[0.9-1.0]
Level of education^d^			
Low	0.6	1.5	0.9
	[0.2-1.7]	[0.6-4.2]	[0.2-4.2]
Intermediate	1.3	1.3	0.5
	[0.7-2.6]	[0.6-2.9]	[0.2-1.2]
High	Reference	Reference	Reference
**Health Belief Model**			
Perceived susceptibility	**1.7**	1.0	**2.9**
	[1.0-2.7]*	[0.6-1.5]	[1.6-5.5] **
Perceived severity	0.6	0.8	1.0
	[0.4-1.0]	[0.5-1.3]	[0.5-2.1]
Cues to action	0.8	**3.3**	**4.7**
	[0.4-1.6]	[1.3-8.3]*	[1.4-15.5]*
Perceived benefits	**9.4**	**22.9**	**150.0**
	[3.6-24.5]**	[7.8-67.7]**	[32.7-688.6]**
Perceived barriers	**0.5**	1.0	0.8
	[0.3-1.0]*	[0.5-2.0]	[0.4-1.4]
Self-efficacy	**10.2**	**12.9**	**10.0**
	[5.6-18.7]**	[7.0-23.8]**	[4.6-22.0]**
**TPB & PMT**			
Subjective norm	1.1	1.6	**1.8**
	[0.7-1.8]	[0.9-2.9]	[1.1-3.1]*
Anticipated regret	0.7	1.1	1.2
	[0.5-1.1]	[0.7-1.8]	[0.7-2.1]
Moral obligation	**3.5**	1.0	1.0
	[1.9-6.5]**	[0.5-1.9]	[0.5-1.8]
Response efficacy	**0.5**	0.6	**0.2**
	[0.3-0.9]*	[0.3-1.1]	[0.1-0.5]**
**Nagelkerke R^2^**	0.6	0.7	0.7

Note: 0 = non-tester; 1 = self-tester

* p < .05; ** *p *< .001

^*a *^Cholesterol testers = 150, non-testers = 183

^*b *^Glucose testers = 185, non-testers = 179

^*c *^HIV testers = 107, non-testers = 174

^d ^Low = primary and secondary school, Intermediate = intermediate vocational education, high = higher vocational education and university

#### Glucose test

Glucose testing was significantly associated with cues to action, perceived benefits, and self-efficacy (Table 3).

#### HIV test

HIV testing was significantly associated with perceived susceptibility, cues to action, perceived benefits, self-efficacy, and subjective norm. However, HIV testers were more likely to have a low level of response efficacy than non-testers (Table 3).

### Differences between testers and non-testers at item level

This section reports all differences between self-testers and non-testers on items that were significant with an alpha below .001. Differences between self-testers and non-testers on items for a test with an alpha below .01 are reported if those differences were significant with an alpha below .001 for the two other tests. An overview of all analyses on item level is available as additional file (see additional file 1: Analyses at item-level).

#### Perceived susceptibility

Glucose testers (p < .001) perceived the chance that they will develop an elevated cholesterol level as higher than non-testers. Cholesterol (p < .001), glucose (p < .001), and HIV testers (p < .01) were more likely than non-testers to indicate that they perceived themselves as being more susceptible to develop elevated cholesterol or glucose levels or to become infected with HIV than others of their age and gender.

#### Perceived benefits

Compared with non-testers, self-testers were more likely to indicate that performing a self-test is important, that self-testing means taking responsibility for one's own health, and that privacy is an important advantage of self-testing (p < .001 for all three tests). Cholesterol (p < .001), glucose (p < .01), and HIV testers (p < .001) were also more likely than non-testers to indicate that self-testing provides a sense of security about one's own health. Time-saving was more likely to be seen as an advantage of self-testing by cholesterol (p < .001), glucose (p < .001) and HIV testers (p < .01) compared to non-testers. Compared with non-testers, HIV testers were more likely to indicate that reassurance is an important advantage of testing (p < .001).

#### Perceived barriers

Non-testers were more likely than cholesterol (p < .001), glucose (p < .01), and HIV testers (p < .001) to indicate that they perceived the costs of self-tests as a barrier.

#### Self-efficacy

Compared to non-testers, self-testers were more likely to disagree with the statements 'performing this self-test is difficult' and 'when performing this self-test I would miss professional assistance' (p < .001 for all three tests), indicating that self-testers have a higher level of self-efficacy. Glucose testers (p < .001) were more likely than non-testers to disagree with the statement 'When interpreting the test result I would miss professional assistance'.

#### Subjective norm

Cholesterol testers (p < .001) were more likely than non-testers to indicate that their partners (or others in their immediate environment) expect from them that they do the test.

#### Anticipated regret

Compared to non-testers, HIV testers (p < .001) were more likely to indicate that they would regret if they did not perform the test and it subsequently appeared that they were HIV-positive.

#### Moral obligation

Cholesterol (p < .001) and HIV testers (p < .001) were more likely to perceive a moral obligation (towards oneself and towards others) to perform the test than non-testers.

#### Response efficacy

Cholesterol testers (p < .001) indicated to have more confidence in the accuracy of an abnormal test result than non-testers. HIV testers (p < .001) were more likely to indicate that they had less confidence in their ability to take a correct subsequent action, in case of an abnormal test result, than non-testers.

## Discussion

The value of self-tests has been heavily debated in the scientific literature. Proponents argue that self-testing increases testing rates, resulting in more timely diagnosis and treatment, self-testing is convenient and provides anonymity, and it promotes patient empowerment [4-7], while opponents hold that at-risk populations do not use self-tests, self-tests entail relatively high costs, and testing without counselling may result in adverse psychological outcomes [4-6]. To the best of our knowledge, however, no research to date has examined the psychosocial determinants of self-test use. In this study, we made a first attempt to investigate the psychosocial determinants of self-test use, specifically for cholesterol, glucose, and HIV tests, among a sample of Dutch Internet users, and to examine whether these determinants are similar across the different tests considered.

As expected, based on our theoretical framework, perceived benefits and self-efficacy are important determinants of cholesterol, glucose, and HIV self-testing. Testers perceived more benefits from performing a self-test than did non-testers. Benefits may function as important beliefs supporting a positive attitude, which is positively associated with intention and behaviour [11]. Alternatively, in view of our operationalization of perceived benefits, testers may perceive self-tests as an important tool to take responsibility for their own health [22]. If responsibility is desired, this may be expressed in self-testing. Additionally, an important motivation for self-testing is the belief that self-testing provides reassurance. In other words, self-testers use a self-test hoping to confirm that they are in good health or have a healthy lifestyle [22]. These results are supported by literature about screening behaviour, which also shows that an important predictor of attending a screening program is the motivation to be reassured [22,23].

Similarly, our results on self-efficacy as a predictor of self-test use are in line with a large body of literature showing that self-efficacy is an important predictor of intentions [15,17,18,24] and screening behaviour [25], which may be considered similar to self-testing in certain ways. Our results show that testers reported significantly higher levels of self-efficacy than non-testers in all three tests considered.

In addition, the results show that gender, perceived susceptibility, cues to action, perceived barriers, subjective norm, moral obligation, and response efficacy may also play important roles, although we did not observe a significant contribution of these factors for all three self-tests.

Whereas gender was a significant predictor of cholesterol self-testing, it was not associated with glucose or HIV self-test use. This could be explained by the fact that 51% of all cholesterol self-tests performed in our sample were street-corner tests, which are mostly carried out in supermarkets. Women are more likely to shop for the groceries in the Netherlands and therefore have easier access to these street-corner tests. Glucose and HIV tests are not offered as street-corner tests in the Netherlands.

Perceived susceptibility was a significant predictor of cholesterol and HIV testing, but not of glucose testing. However, results at item level indicated that glucose-testers indeed perceived themselves as being significantly more susceptible to developing diabetes than non-testers. To explain this contradiction, we tested for collinearity, mediation and moderation, and for interaction and suppressor effects. Unfortunately, none of these could account for the contradictory results. Nevertheless, the results at item level suggest that perceived susceptibility is an important predictor of glucose testing as well.

Results for response efficacy were not consistent and contrary to theoretical predictions. Regression analyses indicated that self-testers had a lower level of response efficacy than non-testers, indicating that self-testers would perceive their response to the self-test result as less effective compared to non-testers, whereas the majority of the analyses at item level indicated the opposite for all three tests. A possible explanation for these contradictory results and the large odds ratio and confidence interval for perceived benefits for HIV testing could be the presence of a suppressor variable, which suppresses irrelevant variance in other predictors, thus overestimating the importance of these predictors. Using the method proposed by Mackinnon and colleagues [26], we did indeed identify a significant suppressor effect for perceived benefits and response efficacy (suppressor effect = 0.73 and 1.09; 95% CI [0.37;1.09] and 95% CI [0.49;1.68], for cholesterol and HIV, respectively). Although respondents seem to value the reliability of the test (as evidenced by the main effect of response efficacy on self-testing behaviour), these are less important as compared to other benefits of self-testing (as evidenced by the fact that the effect of response efficacy is suppressed when adding perceived benefits to the analyses).

An alternative explanation for the unexpected effects of response efficacy for HIV self-testers might be that HIV testers realize that a positive test-result (something is the matter) will not automatically lead to a subsequent action that can change their condition (see additional file 1: Analyses at item-level). In other words, the consequences of a positive test-result have a larger impact on the HIV-testers' response efficacy as compared to the characteristics of the self-test itself.

### Study limitations and strengths

The study had some limitations that should be addressed. First, some items were not optimally operationalized. For instance, cues to action were measured as 'Do you or someone in your immediate environment have elevated cholesterol levels?'. It could be argued that this is one of many possible cues to action, and this single item does not cover the complete construct of cues to action.

Second, our survey was a retrospective study and conclusions about causality thus need to be drawn with caution. As a result of the study design we used, it could be assumed that some of the associations we found might be the result of reverse causality. However, the relations we found are consistent with theoretical predictions provided by the HBM and TPB, which served as a basis for the present study. Furthermore, our results were strengthened by the fact that determinants of self-test use were identified in multiple self-tests and our regression models explained between 61% and 73% of the variance in self-test use. However, to gain more insight into the directionality of the relations between theoretical concepts, a longitudinal study is required.

Third, the present study may have been subject to a selection bias due to the fact that we used an Internet research institute. However, studies on Internet surveys versus paper-and-pencil surveys suggest that Internet surveys may yield similar results as traditional paper-and-pencil surveys [27-29]. Furthermore, the Internet research institute claims that their panel is representative for the Dutch Internet population and since most self-tests are bought via the Internet, we consider the use of this Internet panel as a representative sample of Dutch self-testers.

### Practical implications and future research

Although the pros and cons of self-testing are currently not clear, self-testing is an existing phenomenon which is likely to increase in the future. It is therefore essential that appropriate consumer information will be developed which is tailored to the general and test-specific determinants of a self-test, to provide a solid basis for informed choices about self-testing.

The current study was a first attempt to identify psychosocial determinants that are associated with self-test use and we found an indication that these determinants indeed differ between self-tests. However, more research is needed to identify determinants for self-test use for risk-factors, chronic diseases, and infectious diseases other than cholesterol, glucose, and HIV, to establish if these determinants are indeed test-specific. Furthermore, we need to identify the consumer's information use and needs concerning self-testing, and we have to gain insight in their follow-up behaviour by preferably using qualitative research methods. Currently available consumer information on self-testing needs to be assessed to determine the gaps between the available information and the consumer's needs. Another important issue that needs to be addressed is the level of activation of consumers who are involved in self-testing. Highly activated consumers are more engaged in self-management and are more likely to take the appropriate health actions [30-32]. Insight into these factors is needed to develop appropriate consumer information which ideally includes a decision aid on self-testing to promote informed choice [33-35]. This decision aid needs to be aimed at guiding consumers in their decision process to decide whether they are eligible for the test, and if self-testing is the best test option. Information needs to be tailored to the determinants that are related to self-test use and the consumer's level of activation. By combining and integrating these factors in consumer information on self-testing, consumers are stimulated and guided in making an informed choice about self-testing.

## Conclusions

Despite the limitations of the study, we can conclude that determinants of self-testing cannot be generalized to all self-tests. Perceived benefits and self-efficacy were identified as important determinants of self-testing for three tests but other determinants were more test-specific. Although perceived benefits are strongly associated with self-test use, information about self-testing should also include information about possible disadvantages in order to provide consumers with the opportunity to make informed choices. Additionally, it is very important to inform the general public that self-testing is just one of many possible tools to diagnose a particular disorder or risk factor, and to provide them with information about the possibility of false-positive and false-negative results and the potential psychological and financial consequences of test results.

## Abbreviations

HBM: Health Belief Model; HIV: Human Immunodeficiency Virus; ISO: International Organization for Standardization; PMT: Protection Motivation Theory; TPB: Theory of Planned Behaviour

## Competing interests

The authors declare that they have no competing interests.

## Authors' contributions

JG is involved performing the analyses and drafted the manuscript. NdV and GJD are involved in critically revising the manuscript and provided valuable theoretical and design suggestions. GR and TvdW both conceived of the study, participated in its design and coordination, and helped to draft the manuscript. All authors have read and approved the final version of the manuscript.

## Pre-publication history

The pre-publication history for this paper can be accessed here:

http://www.biomedcentral.com/1471-2458/11/112/prepub

## Supplementary Material

Additional file 1 **Analyses at item-level**. This table shows the results on the analyses at item-level for all three tests under consideration. The table contains the means, standard deviations, test values, degrees of freedom, and p-values for each item. Click here for file
