# Supplementary material for: To test or not to test: A cross-sectional survey of the psychosocial determinants of self-testing for cholesterol, glucose, and HIV
Source: BMC Public Health. 2011 Feb 17;11:112. doi: 10.1186/1471-2458-11-112 (PMC3045947; doi:10.1186/1471-2458-11-112)
Supplement: Additional file 1 — Analyses at item-level. This table shows the results on the analyses at item-level for all three tests under consideration. The table contains the means, standard deviations, test values, degrees of freedom, and p-values for each item. [file 1471-2458-11-112-S1.DOC]

**Table 4.** Differences between testers and non-testers at item level

| **Constructs**  **Items/ answering options^a/b^** | **Cholesterol** | | |  | **Glucose** | | |  | **HIV** | | |
| --- | --- | --- | --- | --- | --- | --- | --- | --- | --- | --- | --- |
|  | **Testers**  **N = 150**  **M (SD)** | **Non-testers**  **N = 183**  **M (SD)** | **Test value**  **[DF]** |  | **Testers**  **N = 185**  **M (SD)** | **Non-testers**  **N = 179**  **M (SD)** | **Test value**  **[DF]** |  | **Testers**  **N = 107**  **M (SD)** | **Non-testers**  **N = 174**  **M (SD)** | **Test value**  **[DF]** |
| **Perceived susceptibility** |  |  |  |  |  |  |  |  |  |  |  |
| 1. Chances | 2.9 (0.9) | 2.8 (0.8) | t = -1.4 [326] |  | 3.0 (1.0) | 2.7 (0.9) | **t = -3.3 [347]***** |  | 1.5 (0.8) | 1.6 (0.7) | t = 0.5  [279] |
| 2. Chances compared to others | 3.2 (0.8) | 2.9 (0.7) | **t = -3.7 [328]***** |  | 3.2 (0.9) | 2.9 (0.8) | **t = -3.2 [347]***** |  | 2.3 (0.9) | 2.0 (0.9) | t = -3.1  [279]** |
| **Perceived severity** |  |  |  |  |  |  |  |  |  |  |  |
| 1. Severity | 4.3 (0.7) | 4.3 (0.6) | t = -0.7 [331] |  | 4.0 (0.7) | 4.0 (0.6) | t = 0.1  [362] |  | 4.8 (0.5) | 4.8  (0.5) | t = -9.2  [279] |
| **Cues to action** |  |  |  |  |  |  |  |  |  |  |  |
| 1. You or someone in environment | Yes = 66.7% | 108  (59.0%) | χ^2^ = 2.7  [1] |  | 163  (88.1%) | 124 (69.3%) | χ^2^ = 19.4  [1]* |  | 21 (19.6%) | 14  (8.0%) | χ^2^ = 8.2  [1]* |
|  | No = 33.3% | 75  (41.0%) |  |  | 22  (11.9%) | 55  (30.7%) |  |  | 86  (80.4%) | 160 (92.0%) |  |
| **Perceived benefits** |  |  |  |  |  |  |  |  |  |  |  |
| 1. Important | 3.6 (0.7) | 3.1 (0.7) | **t = -7.0 [331]***** |  | 3.8 (0.8) | 3.3 (0.7) | **t = -6.4 [362]***** |  | 3.9 (0.7) | 2.9 (0.8) | **t = -10.0**  **[279]***** |
| 2. Responsibility | 3.8 (0.7) | 3.2 (0.8) | **t = -7.2 [328.8] ***** |  | 3.9 (0.7) | 3.3 (0.8) | **t = -6.7 [353.5]***** |  | 3.9 (0.8) | 3.0 (0.9) | **t = -8.6**  **[279]***** |
| 3. Sense of security | 3.5 (0.8) | 3.1 (0.8) | **t = -4.3 [331]***** |  | 3.6 (0.9) | 3.3 (0.8) | t = -3.0 [362]** |  | 3.8 (0.7) | 3.1 (0.9) | **t = -6.9**  **[262.9]***** |
| 4. Fast result | 3.8 (0.8) | 3.8 (0.7) | t = 0.7  [331] |  | 4.1 (0.1) | 4.0 (0.7) | t = -0.7 [362.0] |  | 4.0 (0.7) | 3.7 (0.9) | t = -2.5  [257.3]* |
| 5. Privacy | 3.3 (0.9) | 2.4 (0.8) | **t = - 9.8 [331]***** |  | 3.4 (1.0) | 2.2 (0.8) | **t = -13.5 [343.1]***** |  | 4.0 (0.8) | 2.7 (0.9) | **t = -12.3**  **[246.5]***** |
| 6. Saves time | 3.7 (0.8) | 3.0 (1.0) | **t = -7.3 [330.4]***** |  | 3.8 (0.9) | 2.7 (1.0) | **t = -11.3 [347.1]***** |  | 3.6 (0.8) | 3.3 (1.0) | t = -2.8  [260.1]** |
| 7. Reassurance | 3.6 (0.7) | 3.3 (0.9) | t = -2.5 [329.2]* |  | 3.6 (0.8) | 3.4 (1.0) | t = -2.5 [345.0]* |  | 3.9 (0.6) | 3.6 (0.9) | **t = -3.8**  **[278.7]***** |
| 8. Taking care of health | 3.8 (0.6) | 3.6 (0.8) | t = -2.8 [325.4]** |  | 3.9 (0.6) | 3.8 (0.8) | t = -1.3 [337.7] |  | 3.8  (0.7) | 3.6 (0.9) | t = -2.4  [265.2]* |
| 9. Feels good | 3.8 (0.56) | 3.7 (0.7) | t = -2.5 [330.4]* |  | 3.8 (0.6) | 3.7 (0.8) | t = -1.6 [330.9] |  | 3.8 (0.6) | 3.6 (0.8) | t = -2.7  [277.8]** |
| **Perceived barriers** |  |  |  |  |  |  |  |  |  |  |  |
| 1. Costs | 2.6 (0.9) | 3.3 (1.0) | **t = 7.0 [331]***** |  | 2.8 (1.1) | 3.1 (1.1) | t = 3.0  [362] ** |  | 2.7 (1.0) | 3.5 (1.0) | **t = 6.3**  **[279]***** |
| 2. Too concerned with health | 2.6 (1.0) | 2.9 (1.1) | t = 2.4 [331]* |  | 2.6 (1.0) | 2.7 (1.1) | t = 1.5  [362] |  | 2.5 (0.8) | 2.6  (1.1) | t = 1.4 [263.0] |
| 3. Too concerned scaring | 2.5 (1.0) | 2.6 (1.0) | t = 0.4  [331] |  | 2.5 (0.9) | 2.6 (1.0) | t = 0.3 [354.3] |  | 2.8 (1.0) | 2.7  (1.1) | t = -0.8  [279] |
| 4. Thinking about testing scaring | 2.0 (0.7) | 2.0 (0.8) | t = 0.0  [331] |  | 1.9 (0.7) | 2.0 (0.9) | t = 1.5 [337.3] |  | 2.2 (0.8) | 2.1 (0.9) | t = -0.3  [279] |
| 5. Thinking about testing insecure | 2.0  (0.7) | 2.0  (0.8) | t = 1.0  [331] |  | 2.0 (0.8) | 2.1 (0.9) | t = 1.4 [345.2] |  | 2.3 (0.8) | 2.2  (0.9) | t = -0.8  [279] |
| **Self-efficacy** |  |  |  |  |  |  |  |  |  |  |  |
| 1. Difficult | 3.7 (0.7) | 2.7 (1.0) | **t = -10.6 [324.6]***** |  | 4.0 (0.7) | 2.6 (1.0) | **t = -15.4 [333.4]***** |  | 3.4 (0.7) | 2.0 (1.0) | **t = -12.5 [279]***** |
| 2. Performing professional guidance | 3.5 (0.8) | 2.4 (1.0) | **t = -11.4 [330.8]***** |  | 3.7 (0.9) | 2.2 (1.0) | **t = -15.3 [362]***** |  | 3.3 (0.8) | 2.2 (1.0) | **t = -9.6 [279]***** |
| 3. Interpreting professional guidance | 3.3 (0.9) | 3.0 (1.0) | t = -2.6 [331]** |  | 3.6 (0.9) | 3.1 (1.0) | **t = -4.3 [362]***** |  | 3.2  (0.9) | 3.2 (0.9) | t = -0.2  [279] |
| **Subjective norm** |  |  |  |  |  |  |  |  |  |  |  |
| 1. Expectations environment | 2.3 (1.0) | 2.0 (0.9) | **t = -3.1 [303.2]***** |  | 2.1 (1.0) | 2.0 (0.9) | t = -1.1 [354.2] |  | 2.6 (1.0) | 2.3 (1.0) | t = -2.2  [279]* |
| **Anticipated regret** |  |  |  |  |  |  |  |  |  |  |  |
| 1. Regret afterwards | 3.4 (0.9) | 3.1 (1.1) | t = -2.8 [331]** |  | 3.5 (1.1) | 3.2 (1.1) | t = -2.5 [362]* |  | 4.0 (1.0) | 3.5 (1.2) | **t = -4.1**  **[253.6]***** |
| **Moral obligation** |  |  |  |  |  |  |  |  |  |  |  |
| 1. Moral obligation to self | 3.1 (0.9) | 2.5 (1.0) | **t = -6.3 [328.9]***** |  | 3.0 (1.0) | 2.7 (1.0) | t = -2.6 [362]* |  | 3.6 (0.9) | 3.0 (1.1) | **t = -4.8 [279]***** |
| 2. Moral obligation to environment | 2.9 (1.0) | 2.4  (0.9) | **t = -5.0 [331]***** |  | 2.7 (1.0) | 2.5 (1.0) | t = -2.2 [362]* |  | 3.5 (0.9) | 3.0 (1.1) | **t = -4.0 [258.7]***** |
| **Response efficacy** |  |  |  |  |  |  |  |  |  |  |  |
| 1. Reliability | 3.6 (0.7) | 3.5 (1.1) | t = -1.6 [317.3] |  | 3.9 (0.7) | 3.7 (0.9) | t = -1.9 [327.7] |  | 3.8 (0.8) | 3.5 (1.2) | t = -2.2 [278.0]* |
| 2. Normal result | 3.6 (0.7) | 3.6 (1.0) | t = -0.1 [318.7] |  | 3.8 (0.7) | 3.8 (0.8) | t = 0.3 [347.3] |  | 3.8 (0.8) | 3.8 (1.0) | t = -0.2  [279] |
| 3. Abnormal result | 3.6 (0.7) | 3.3 (1.1) | **t = -4.0 [309.6]***** |  | 3.6 (0.8) | 3.5 (1.0) | t = -1.6 [339.2] |  | 3.5 (0.8) | 3.5 (1.1) | t = 0.1 [264.5] |
| 4. Subsequent action | 3.9 (0.7) | 3.8 (0.9) | t = -1.0 [330.1] |  | 4.0 (0.6) | 4.0 (0.8) | t = -0.4 [330.4] |  | 3.8 (0.7) | 4.1 (0.9) | **t = 3.2**  **[279]***** |

^a^ Abbreviated items correspond to the complete items in Table 1

^b^ Answering options correspond to answering options provided in Table 1

*Note:* * < .05, ** < .01, *** < .001
